# A Novel Approach to Evaluating Mobile Smartphone Screen Time for iPhones: Feasibility and Preliminary Findings

**DOI:** 10.2196/11012

**Published:** 2018-11-19

**Authors:** Aubrey D Gower, Megan A Moreno

**Affiliations:** 1 Department of Pediatrics School of Medicine and Public Health University of Wisconsin - Madison Madison, WI United States

**Keywords:** smartphone, youth, mobile apps, mobile phone, screenshot

## Abstract

**Background:**

Increasingly high levels of smartphone ownership and use pose the potential risk for addictive behaviors and negative health outcomes, particularly among younger populations. Previous methodologies to understand mobile screen time have relied on self-report surveys or ecological momentary assessments (EMAs). Self-report is subject to bias and unreliability, while EMA can be burdensome to participants. Thus, a new methodology is needed to advance the understanding of mobile screen time.

**Objective:**

The objective of this study was to test the feasibility of a novel methodology to record and evaluate mobile smartphone screen time and use: battery use screenshot (BUS).

**Methods:**

The BUS approach, defined for this study as uploading a mobile phone screenshot of a specific page within a smartphone, was utilized within a Web-based cross-sectional survey of adolescents aged 12-15 years through the survey platform Qualtrics. Participants were asked to provide a screenshot of their battery use page, a feature within an iPhone, to upload within the Web-based survey. Feasibility was assessed by smartphone ownership and response rate to the BUS upload request. Data availability was evaluated as apps per BUS, completeness of data within the screenshot, and five most used apps based on battery use percentage.

**Results:**

Among those surveyed, 26.73% (309/1156) indicated ownership of a smartphone. A total of 105 screenshots were evaluated. For data availability, screenshots contained an average of 10.2 (SD 2.0) apps per screenshot and over half (58/105, 55.2%) had complete data available. The most common apps or functions included Safari and Home and Lock Screen.

**Conclusions:**

Study findings describe the BUS as a novel approach for real-time data collection focused on iPhone screen time and use among young adolescents. Although feasibility showed some challenges in the upload capacity of young teens, data availability was generally strong across this large dataset. These data from screenshots have the potential to provide key insights into precise mobile smartphone screen use and time spent per mobile app. Future studies could explore the use of the BUS methodology on other mobile smartphones such as Android phones to correlate mobile smartphone screen time with health outcomes.

## Introduction

Smartphones and their vast functionalities have become an integral part of individuals’ lives today [1]. In the United States alone, smartphone ownership has increased to 77%, with 95% of teens aged 13-17 years reporting access to or ownership of a smartphone (92%) [2,3]. Globally, similar trends are seen in smartphone ownership, including among emerging economies [4]. Among adolescent smartphone owners, 64% reported “everyday use” in a recent survey [5]. As ownership and accessibility to mobile smartphones have become ubiquitous, so has the need for research into the implications, such as positive and negative health consequences. Recent research suggests high screen media usage is associated with poor sleep and diminished academic performance among adolescents and adults [6-8]. Thus, accurate and feasible methodologies to study mobile screen time are necessary to further understand these relationships.

Previous methodologies to understand mobile screen time have typically relied on traditional self-report and cross-sectional research design [6-10]. However, self-report is vulnerable to systematic and confounding bias [11]. Previous work has shown conflicting results related to media use time with the use of self-report. Some studies have found self-report app usage to underestimate app and smartphone usage [12]. On the other hand, studies have also found self-report by participants to overestimate Web-based time [13,14]. These inconsistencies have led some researchers to suggest that self-reported smartphone use should be interpreted with caution [15].

Another approach to evaluate mobile media use is ecological momentary assessment (EMA). In this approach, participants are typically contacted multiple times per day to report their real-time screen use [16,17]. While this approach improves upon self-report biases, such as recall bias, it can be highly burdensome to participants [18]. In addition, these methodologies often fail to obtain large-scale and representative samples due to costs to researchers to provide the compensation necessary to attract participants [19]. Thus, a new methodology is needed to advance the assessment of mobile screen time that not only improves accuracy compared with self-report but is also not burdensome to participants.

One possible approach to understanding mobile smartphone screen time while limiting participant burden is to leverage passive tracking that smartphones are programmed for via battery use reporting. Most smartphones, such as iPhones, track battery use per app and phone function (eg, Home and Lock Screen). A majority of smartphones will then report this battery use by time, including both active onscreen and background use. The “battery use” function and display, thus, serve as an indicator for real-time app and smartphone activity.

In this research protocol, we present preliminary findings of the battery use screenshot (BUS) approach among young adolescents. The objective of this study was to test the feasibility of the BUS approach to obtain and evaluate iPhone screen time data.

## Methods

### Design

Data for this study were collected as a planned protocol evaluation as part of a larger study related to technology rules and health behaviors among adolescents aged 12-15 years. The BUS approach was defined for this study as the upload of a mobile phone screenshot of the “battery use” page within the participant’s smartphone. To assess the feasibility of the BUS approach, this study was designed as a cross-sectional Web-based survey of 12- to 15-year-old adolescents. We collected data between June and July 2017 using the Qualtrics survey platform. The study was approved by the University of Washington Institutional Review Board.

### Participants and Recruitment

Youth who were between the ages of 12 and 15 years and who could read English were eligible to complete the survey. As described in previous studies [20], Qualtrics draws upon previously established age panels within their Web-based database, allowing for targeted recruitment. Eligibility was assessed prior to beginning the Web-based informed consent process. Once informed consent from parents was obtained, participants could begin the survey. Compensation was provided to participants through Qualtrics for survey completion.

### Data Collection Process

#### Variables

##### Phone Ownership

Participants were asked whether they currently had a smartphone capable of taking a screenshot. Participants were also asked to indicate whether the phone was their own, their parent’s smartphone, or that they did not own a smartphone.

##### Demographics

Age, gender, parental education, and race or ethnicity were assessed.

#### Screenshot Upload

**Figure 1:**
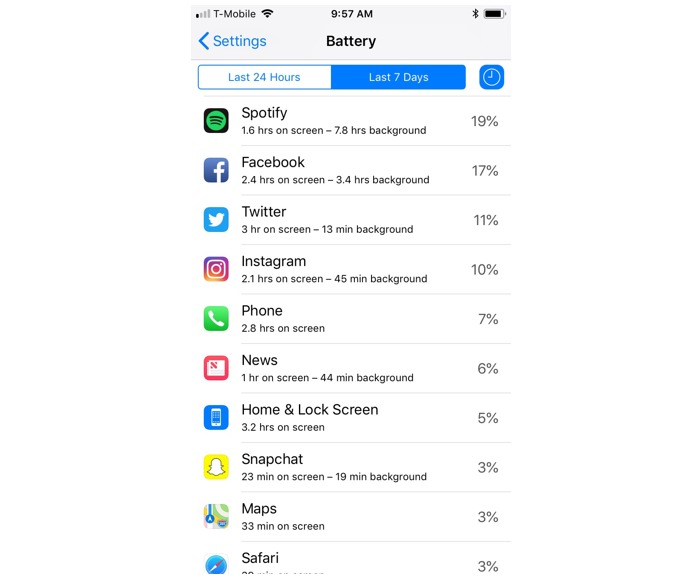
Example of an iPhone battery use screenshot from an Apple iPhone 6 with IOS 11; image was produced by first author.

Participants were provided an example BUS within the survey (Figure 1) and then asked to create their own screenshot from the phone they currently used, if they had one. Instructions included first asking the participant to go to the “Settings” section of their smartphone and clicking the “Battery” option. Once in the “Battery” section, participants were asked to locate the “battery usage” data displayed. For iPhones, participants were asked to click both the “Last *x* days” and the clock symbol in the right-hand corner to display both onscreen and background activity for each app listed. Participants were asked to compare their display to the example provided to ensure the correct data were viewable. Participants were then asked to screenshot this display by holding down the home and power button at the same time to capture the image. Once the screenshot was captured, participants were asked to upload the image, as a .jpg or .png, to a file dropbox within the survey.

### Analysis

Descriptive statistics were used to analyze feasibility and data availability. The most commonly used apps were determined based on their frequency among the evaluated screenshots. Analysis focused on iPhone screenshots because of inconsistency among Android platforms in the display of battery usage. Screenshots were excluded if they contained anything other than the battery use page from a personal phone.

### Outcomes

#### Feasibility

Measures to assess feasibility included a survey question asking about personal smartphone ownership and response rate to BUS upload request.

##### Data availability

To characterize data availability, content analysis assessed apps or functions per screenshot and completeness of the screenshot including display of “onscreen” and “background” time and displayed percentage of battery use per app. Functions were characterized as iPhone functions such as Home and Lock Screen that use battery life but are not considered typical downloadable apps. The five most commonly used apps or functions among evaluated screenshots were assessed. The most used apps or functions for each screenshot were defined based on displayed battery use percentage.

## Results

### Demographics

A total of 1156 adolescents with an average age of 13.6 (SD 1.09) years responded to the survey. The overall survey sample comprised 49.48% (572/1156) females and 72.84% (842/1156) Caucasian people. Among the sample of participants for which screenshots were evaluated, 52.3% (79/151) were females and 75.5% (114/151) were Caucasian people. Full demographics for both populations can be seen in Table 1.

### Feasibility

**Figure 2:**
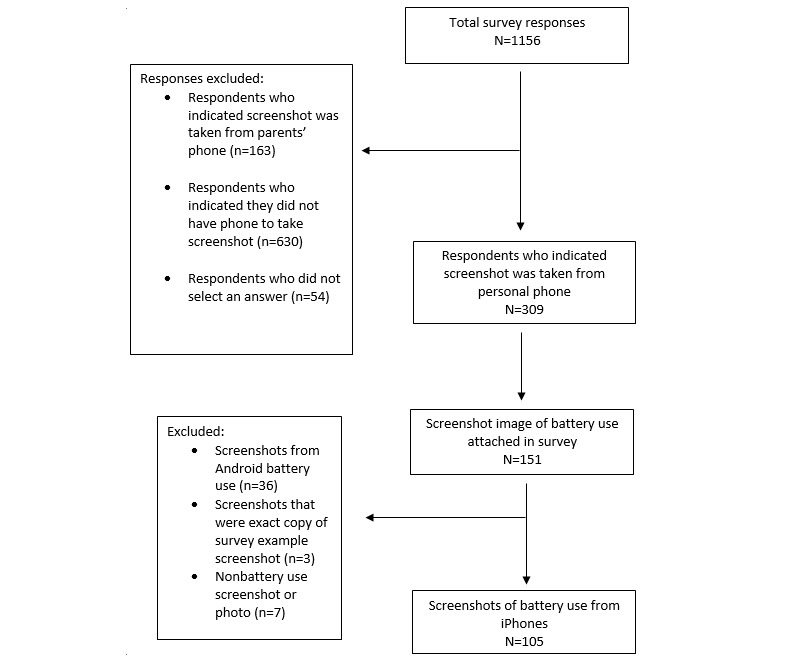
Protocol results flowchart.

Among the overall survey sample, 26.73% (309/1156) indicated they had their own phone. Among these adolescents, 48.9% (151/309) completed the BUS upload request (Figure 2).

**Table 1:** Demographic characteristics of participants.

Characteristics		Total survey sample (N=1156)	Sample screenshots evaluated (n=151)
Age in years, mean (SD)		13.6 (1.09)	13.7 (1.05)
**Gender, n (%)**			
	Female	572 (49.48)	79 (52.32)
	Male	573 (49.57)	69 (45.70)
	Female to male transgender people	4 (0.35)	2 (1.32)
	Male to female transgender people	0 (0.00)	0 (0.00)
	Not sure	3 (0.26%)	1 (0.66)
**Race or ethnicity, n (%)**			
	White or Caucasian	842 (72.84)	114 (75.50)
	Black or African American	80 (6.92)	12 (7.95)
	Hispanic or Latino	101 (8.74)	12 (7.95)
	Asian	56 (4.84)	6 (3.97)
	American Indian or Alaska Native	8 (0.69)	0 (0.00)
	Native Hawaiian or Pacific Islander	4 (0.35)	1 (0.66)
	More than one race	41 (3.55)	4 (2.65)
	Other	10 (0.87)	2 (1.32)
**Parental education level, n (%)**			
	High school Graduate	118 (10.21)	9 (5.96)
	Tech school or associate degree	88 (7.61)	10 (6.62)
	Some college	122 (10.55)	16 (10.60)
	College degree	361 (31.23)	56 (37.09)
	Some graduate school	39 (3.37)	11 (7.28)
	Completed graduate degree	299 (25.87)	37 (24.50)
	Other	116 (10.03)	12 (7.95)
	Did not answer	13 (1.12)	0 (0.00)

### Data Availability

A total of 105 iPhone screenshots, received as .jpg or .png images, were used for the evaluation of data availability. Each screenshot contained an average of 10.2 (SD 2.00) apps. More than half (58/105, 55.2%) had complete data availability, indicating successful implementation of instructions provided. Complete screenshots allowed for the ability to view the apps used, battery use percentage per app, and total minutes or hours of both “background” and “on screen” usage (Figure 1).

### Most Commonly Used Apps

**Figure 3:**
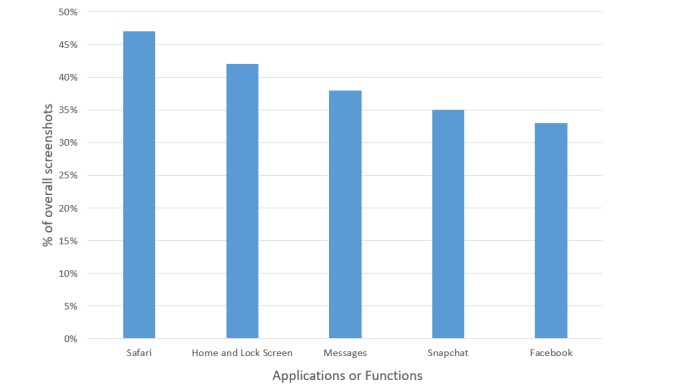
Most common apps or functions among analyzed screenshots.

Figure 3 shows the frequency of the most common apps or functions to appear in the five most used apps or functions among the analyzed screenshots. Safari, an internet search engine, was the most common app among screenshots; 46.7% (49/105) of screenshots included Safari within the five most used apps.

## Discussion

### Principal Findings

This study describes a novel BUS approach to test feasibility, data availability, and most common apps among a young adolescent population. The inclusion of the BUS approach within a Web-based survey adds the capacity to assess mobile smartphone screen time that is accessible for both participants and researchers in a highly technological environment. Battery use is automatically monitored by the operating systems of most smartphones [21], reducing both the bias of self-report as well as burden on participants to record real-time smartphone and app use. Compared with typical EMA approaches, this methodology can improve the capacity to deliver real-time data as well as allow for data collection from larger, Web-based samples [22].

The feasibility of our approach was mixed with both strengths and challenges. Among the participants who did upload a screenshot, the images provided clear data that could be viewed, stored in a deidentified manner, and categorized for further analysis. Our challenges in feasibility may be explained by the rate of phone ownership in our young adolescent sample, which is lower than recent reports of 56% smartphone ownership among youth aged 8-18 years [23]. Among adolescents with their own phone, approximately half were willing or able to upload a screenshot. It is possible that a study population of older adolescents and adults may have higher phone ownership and better understanding and capacity to upload a screenshot. An additional explanation for this challenge may be the survey platform Qualtrics, which allows users to take surveys on both mobile phones and desktop computers. Participants who used a desktop computer to complete the survey may have experienced an additional burden in uploading a screenshot from a different device, which may have contributed to the overall feasibility of this study. Compensation of US $13 was determined by Qualtrics based on the initial suggested sample size for the larger study. Based on our results, low monetary compensation may be a factor in willingness to complete the screenshot task and should be considered when incorporating the BUS approach into future studies.

Over half of the screenshots analyzed contained complete data including the display of “onscreen” versus “background” time per app. One possible reason for cases of incomplete data may be that participants had an older iOS operating system, as the battery usage feature is only available on iOS 9 and newer operating systems [24]. This should be taken into consideration in future studies utilizing this methodology in iPhones. In addition, this study relied on a single screenshot, which may not have captured total app usage. To strengthen overall data availability, future studies might require as many screenshots as necessary to provide the full range of apps used by a participant.

While our study illustrates both strengths and challenges to the proposed research protocol, it serves as a valuable starting point for considering how to advance data collection methods to understand mobile smartphone screen time and media use in iPhones. Studies have concluded that smartphone apps can be beneficial in monitoring and evaluating patients [25-27] as well as increasing adherence to medical interventions [28,29]. The BUS approach offers the ability to take these studies further in understanding real-time use and overall time spent on apps as a factor that could contribute to health outcomes. This methodology offers a framework that could be adapted to Android phones, which offer varying displays of battery use per app and time spent per app. When data and display of battery use are available, a BUS approach may still be utilized as a data collection process. Instructions for the screenshot would need to be adjusted to reflect the varying battery use displays of Androids, and further exploration of these displays should be used to inform these instructions. By expanding the BUS approach to Android devices, future studies would also allow for greater sampling of socioeconomic status, with lower socioeconomic status individuals and families more likely to own Android devices.

The BUS approach may also be combined with a variety of research methodologies including Web-based cross-sectional surveys, as was done in this study, or used as a tool for monitoring smartphone app use longitudinally. In a previous pilot study conducted using BUS, older adolescent iPhone users were asked to submit weekly screenshots of battery use for 9 weeks, with a 94% retention rate over 5 weeks and 60% retention rate over 9 weeks [30]. A further advantage of the BUS approach is the ability to collect comprehensive data related to mobile smartphone screen time without the need for an additional app or programming. Thus, this methodology is accessible for researchers without the means for software or app development.

### Limitations

There are limitations to this feasibility study. The sample size of screenshots analyzed is limited and is not representative of the larger adolescent population. The sample overall may not be representative of the US racial or ethnic makeup for youth aged under 18 years because current US census records differ from our study’s demographics and estimate this population to be 51% white, 15% black or African American, and 25% Latino people [31]. In addition, the battery use page only displays phone use while an iPhone is not charging and may not account for the full time spent using a smartphone device. Furthermore, adolescents may have access to other phones, including their parents’, which may underestimate overall screen time. Finally, in this feasibility study, only iPhone screenshots were evaluated. Results may not generalize to all smartphones or mobile devices with battery use tracking. We did not test the BUS tracking method against other methods of tracking app use to assess its accuracy.

### Conclusion

Though feasibility with the BUS methodology showed challenges in phone ownership rates and upload capacity of young teens, availability of data was generally strong across this large dataset. The data available from screenshots have the potential to provide key insights into precise mobile smartphone screen use and amount of time spent per mobile app. The BUS approach may provide an innovative and complementary approach to understanding smartphone screen use without the need for complex programming or mobile app development. Future studies could improve upon the BUS methodology to correlate mobile smartphone screen time with health outcomes.

## Abbreviations

BUS: battery use screenshot
EMA: ecological momentary assessment
